# Registration of Midwives and Nurses

**Published:** 1889-12-14

**Authors:** 


### The Hospital

A Weekly Journal of

Science, Medicine, Nursing, and Philanthropy.

*For Hospitals, Asylums, Medical Practitioners, Students, Nurses, Families, Parishes, Congregations, and the Charitable Public.*

Vol. VII. No. 168.] [December 14, 1889.

### Registration of Midwives and Nurses.

In another column a full report will be found of an interesting discussion and the decisions arrived at on this question by the General Council of Medical Education and Registration of the United Kingdom. The brief note which we have appended to the report of the proceedings will be a sufficient explanation of the position of the question when brought before the council. The subject was raised in consequence of a correspondence between Mr. Henry Fell Pease, M.P., and Prof. Marshall, the president of the council.

Mr. Pease had given notice of a Bill for the registration of midwives, and he was anxious to ascertain the position which the Medical Council were prepared to take in the matter. The questions he raised were: Would the Medical Council undertake the duty of registering midwives providing it was conferred on them by statute? If not, what attitude would they assume towards a board of registration, if established with full power to do everything which was necessary to protect the interests of the public and the midwife properly so called? Mr. Pease urged that the proposed board should be a board of registration for midwives in the same sense as the General Medical Council is a board of registration for doctors. He desired to know whether the council thought it necessary or even desirable to establish any further examining body or bodies for granting certificates to midwives in addition to the Obstetrical Society, the various lying-in hospitals of repute, and the school of the Rotunda Hospital at Dublin. Was it most desirable to nominate certain persons specifically, such as the President of the Medical Council, the Presidents of the Royal Colleges of Physicians and Surgeons in London, Edinburgh, and Dublin, and the principals of the various British universities, or would the Medical Council itself undertake the duty of nominating members to serve on the board of registration for midwives? Ought not the Bill to apply to Ireland and Scotland as well as to England and Wales? Mr. Pease further pointed out that the desire of members on both sides of the House, who were interested in the question, was to draft the best possible Bill, and so to settle this question adequately and at once. We state these facts as the best possible answer to those who maintain that the Midwive's Institute desires to remove the control of midwives out of the hands of the medical profession. On the contrary, it will be seen that the object of Mr. Pease and his supporters is to enact that the registration of midwives shall be dealt with solely by the professional authorities themselves.

Mr. Pease dealt only with the registration of midwives, but a memorandum was also received by the Medical Council from the British Nurses' Association, which desires to establish under the same board a register for nurses as well as for midwives.

This caused the question of registration for nurses and midwives to be discussed by the Medical Council, with the result that that body endorsed the views held by Sir William Turner that to go into the question of nurses would be to exceed the powers of the Medical Council, because it was a Council of Medical Education and Registration and not a nursing institution. We have always maintained it was impossible to treat midwives and nurses on the same basis, seeing that the work done by these two classes of women is entirely distinct and wholly different in character. Besides, the social condition and character of nurses as a body are far superior to those of midwives under existing circumstances. On the first point Sir Walter Foster showed "that it was a growing scandal to civilisation that they should have independent persons taking charge of women in perilous conditions, and should have a considerable amount of disease brought about by their ignorance. The very last case of puerperal fever he had seen was found to have arisen from a midwife having occasionally left her patient to go into another house to attend a child suffering from a malignant form of sore throat. This showed how the ignorance of midwives was really a peril to the community, and, that peril falling upon the poorest classes, it was a matter of public interest that some steps should be taken to prevent it. To that end it was essential that these women should be educated in some form, and that when educated they should be under supervision and restraint." It must be obvious to the most superficial observer, from these statements, that the dangers arising to a patient from the work of an incapable midwife are far greater than any dangers arising from similar incapacity on the part of a nurse. No one technically acquainted with the respective duties of nurses and midwives would for one moment class them together, or advocate their being treated on the same basis in regard to registration and discipline.

The dual board advocated by the British Nurses' Association was thus shown to be dangerous and impossible; and Dr. Atthill, whose experience of midwives is greater, probably, than that of any other living man, showed clearly that no one who was really anxious to raise the status of trained nurses could consent to group them with midwives under present conditions. Dr. Atthill stated that "the Rotunda Hospital licensed annually a large number of women as midwives, and the greatest possible care was taken that these women were qualified. Notwithstanding all this, it was very hard to get a good mid wife, the applicants consisting of servants, who, having been for many years in service, got tired of that occupation, and came up for training, the fees being paid by their former mistresses. Other women were sent up by Poor Law guardians, many of whom were not able to read or write. If this was the experience in Ireland what would it be in the country at large, where there was no such system as had for several years been in existence in Ireland. Some women who came to the Rotunda were well educated and desired to make midwifery their profession, and they turned out exceedingly competent." Dr. Leishman gave similar testimony. Such evidence as this will open the eyes of the whole nursing body and of all who take an interest in their welfare to the impossibility of their consenting to be classed with midwives either socially, professionally, or in a common association for their mutual intercourse and protection. This was fully recognised by the Medical Council, which declined on a division to accept the proposals of the British Nurses' Association, or to entertain the question of a dual register under one board. They further declined to consider the question of the registration of nurses, because, as Sir John Simon said, the subject of midwives was forced upon the council by Parliament, and was ripe for legislation, but this other question was not.

It is matter for congratulation that the scheme of the Midwive's Institute should have been thus supported by the Medical Council, and great credit is due to Mr. Fell Pease tor the statesmanship he has displayed in referring the subject to them for consideration and decision. It will be seen that the Medical Council declared that they regarded the absence of public provision for the education and supervision of midwives as productive of a large amount of grave suffering and fatal disease among the poorer classes, and they therefore urged upon the Government the importance of passing into law some measure for the education and registration of midwives. Nothing could be more conducive to the public interest than this decision of the Medical Council, and we are glad to think that there is every prospect of an adequate Bill for the regulation of midwives receiving the sanction of the Legislature during the next session of Parliament.

